# Exploring the Relationship Between Serum Creatinine and Salivary Creatinine Levels in Patients With Chronic Kidney Disease in South India: A Cross-Sectional Study

**DOI:** 10.7759/cureus.57709

**Published:** 2024-04-06

**Authors:** Joshua Chacko, Raymond Haward, Aiswarya Roy Karintholil, Joel Sabu, Glenn Austin Fernades

**Affiliations:** 1 Internal Medicine, Father Muller Medical College, Mangaluru, IND; 2 Internal Medicine, Vydehi Institute of Medical Sciences and Research Centre, Bengaluru, IND

**Keywords:** cross sectional studies, electrolyte imbalance, urea, phlebotomy in elderly, gfr declining, chronic kidney disease (ckd), creatinine baseline, urine creatinine

## Abstract

Background

In this study, researchers investigated non-invasive methods for analyzing creatinine levels by using saliva to address the need for frequent phlebotomy in chronic kidney disease (CKD) patients, which can damage their veins due to repeated blood withdrawals for creatinine level assessments.

Methods

This is a cross-sectional study in a tertiary healthcare setting conducted on 50 patients diagnosed with CKD. After collecting serum and salivary creatinine, we used Pearson correlation to assess the correlation between the two factors.

Results

The mean age of the patients was 50 years with a standard deviation of ± 15.32 years. 33 (66%) patients were males and 17 (34%) were females. Most patients were in the age group of 51 - 70 years, comprising 26 (52%) of the sample. The serum creatinine and salivary creatinine values ranged between 7.26-12.00 and 0.45-0.98, respectively. The median values were 9.72 and 0.75, respectively. There was a very weak positive linear relationship between serum and salivary creatinine levels; however, there was no significant association between them (p = 0.52). Nonetheless, a statistically significant, moderately negative linear correlation exists between serum urea and serum albumin (r = -0.36; p = 0.01). Additionally, there is a statistically significant weak negative linear correlation between serum chloride and serum urea (r = -0.3; p = 0.03). Comparing serum chloride and serum sodium reveals a statistically significant, moderately positive linear relationship (r = 0.4; p = 0.004). Serum phosphorus and serum creatinine display a statistically significant moderate positive linear relationship (r = 0.44; p = 0.001). Moreover, estimated glomerular filtration rate (eGFR) and serum creatinine exhibit a statistically significant strong negative linear correlation (r = -0.79; p < 0.001), while eGFR and serum phosphorus demonstrate a statistically significant weak negative linear correlation (r = -0.30; p = 0.03).

Conclusion

The study found no significant association between salivary and serum creatinine levels. Further multicentric studies on a larger population must be conducted to find the potential correlation between serum and salivary markers.

## Introduction

Chronic kidney disease (CKD) is a major health problem worldwide. It happens when your kidneys gradually stop working well. The current guidelines indicate a glomerular filtration rate (GFR) of less than 60 ml/min per 1.73 m2 for 3 months or more [1]. This can lead to various health issues, from mild problems to serious kidney failure. CKD needs meticulous management to slow down its progress and prevent complications.

The main reason behind CKD is diabetes, both type 1 and type 2 [2]. Other causes include long-term high blood pressure, infections that affect the kidneys, certain diseases that cause kidney inflammation, genetic conditions like polycystic kidney disease, blockages in the urinary tract, kidney infections, certain medications, autoimmune diseases like lupus, birth defects in the urinary tract, toxins, heavy metals, and pollution, which can also lead to CKD [2].

CKD can bring lots of problems like heart disease, anemia, weak bones, pain in bones, fractures, calcium buildup in tissues, problems with fluids and minerals in the body, a buildup of waste products in the blood, high blood pressure, a weak immune system, nerve damage, dyslipidemia, left ventricular failure, hyperkalemia, hypophosphatemia, and slower healing of wounds [3].

Assessing the GFR is crucial for diagnosing and staging CKD. We can use different markers like inulin, creatinine, urea, and cystatin C for this purpose [4]. Clinical settings commonly prefer creatinine for GFR measurement due to its easy-to-obtain nature, cost-effectiveness, practicality, and consistency when compared to other markers. However, drawing blood for serum creatinine can be uncomfortable for patients and may lead to complications such as bruising, bleeding, infection, nerve damage, fainting, and, in rare cases, arterial puncture or hematoma formation [5]. Patients suffering from clotting factor deficiency from hemophilia faced difficulty following compliance with routine phlebotomy [6]. Also, CKD patients on dialysis who have hepatitis B and C can increase the risk of HCWs getting hepatitis from needle stick injuries. Hence, researchers seek non-invasive tests that avoid these complications [6].

Salivary glands produce saliva, which contains electrolytes, enzymes, mucins, antimicrobial agents, and antibodies, in addition to creatinine [7, 4]. Utilizing saliva proves advantageous due to its simplicity, ease, non-invasiveness, cost-effectiveness, and repeatability [6]. In patients with CKD, elevated levels of blood urea nitrogen (BUN) and creatinine passively diffuse into saliva [8]. Studies conducted by Temilola et al. demonstrated a positive correlation between salivary and serum creatinine levels [9]. Building upon this foundation, our study aimed to compare salivary and serum markers in CKD patients.

## Materials and methods

This cross-sectional study was conducted at a tertiary care center in Mangaluru, in Karnataka state in South India, for 3 months on CKD patients. After obtaining ethical committee approval from the Father Muller Institutional Ethics Committee (FMIEC), Father Muller Medical College, Mangalore, India (approval number: FMIEC/CCM/413/2020), the study included 50 patients who were 18 years of age or older. Anonymity and confidentiality were maintained by not including the names of the participants, and the data was not accessible to anybody except the researchers and the statistical support unit. Written, informed consent was obtained from the participants. Participants had the right to refuse to participate at any point in the study.

Blood & salivary sampling

In this study, blood samples were obtained from the antecubital veins of the arm using serum separator tubes, yielding 2 mL of blood. The collected blood samples were allowed to clot at room temperature for one hour. Subsequently, they were centrifuged at 1000 g for 10 minutes at 4°C.

As for saliva collection, participants were asked to provide 2 mL of whole saliva. They were instructed to sit comfortably with their eyes open, tilting their heads slightly forward. Saliva was accumulated in a sterile container by spitting every minute or just before feeling the urge to swallow. This process continued until 2 mL of saliva was collected. Participants were advised to refrain from eating and drinking for at least 90 minutes prior to collection and to rinse their mouths thoroughly with water beforehand.

After saliva collection, samples were centrifuged at 1000 rpm for 10 minutes. The resulting supernatant was then stored at -80°C before final analysis. Creatinine levels in both saliva and serum samples were analyzed using a Roche Cobas® 6000 analyzer (Roche Diagnostics Corporation, Indianapolis, USA). This comprehensive procedure ensured the proper collection and processing of both blood and saliva samples for subsequent analysis.

Inclusion criteria

Adult patients who had a diagnosis of CKD, who were 18 years of age or older, both male and female, and who were ready to provide informed consent were included in the study.

Exclusion criteria

Patients without CKD, with the presence of oral pathology, bleeding from the mouth, pregnant women, with recent trauma, with a recent history of infusions, and with hospitalizations were excluded from the study.

Statistical analysis

Statistical analysis was performed and analyzed using IBM Statistical Package for Social Sciences (SPSS) version 17 (IBM Corp., Armonk, USA) after entering the obtained data in a Microsoft Excel spreadsheet (Microsoft Corporation, Redmond, USA). We used Spearman's correlation coefficient (r) to test the correlation between serum and salivary creatinine levels.

Sample size

The sample size was determined using the formula n = 2 (Zα ​⋅ Zβ​)^2 . ^σ^2^ / d^2^ , where Zα ​= 1.96 for a 95% confidence interval and Zβ = 0.84 for 80% power. The minimum required sample size was found to be 45.

## Results

The study consisted of 50 participants with a mean age of 53.90 years and a standard deviation of ± 15.32 years. Most of the participants' age group fell under 51-70 years (Table 1).

**Table 1:** The demographic profile of the study participants The term 'n' represents the total number of patients. The demographic profile showed that the majority of subjects belonged to the age group of 51–70 years old (52%), and most of the participants were male (66%).

Demography	Variable	Frequency (n = 50)	Percentage
Age in years	18-30	6	12
	31-50	11	22
	51-70	26	52
	>70	7	14
Gender	Female	17	34
	Male	33	66

Table 2 provides serum creatinine and salivary creatinine values, and their range lies between 7.26-12.00 and 0.45-0.98, respectively. The median values are 9.72 and 0.75, respectively. It also represents the serum calcium, albumin, urea, sodium, potassium, chloride, phosphorous, and eGFR values.

**Table 2:** Summary of laboratory values for renal and electrolyte parameters in a population: mean, median, and interquartile range. The table provides mean values with standard deviations and medians for serum parameters, alongside the 25th and 75th percentiles to illustrate data spread. The 25th percentile denotes the value below which 25% of data falls, while the 75th percentile signifies the value below which 75% of data falls. These percentiles offer insights into data distribution, aiding in understanding the variability of the observed serum values.

Lab values	Mean±SD	Median	Interquartile range	
			25th percentiles	75th percentiles
Serum creatinine	9.50±3.05	9.72	7.26	12
Salivary creatinine	0.83±0.60	0.75	0.45	0.98
Serum calcium	8.60±1.00	8.44	8.1	9.01
Serum albumin	4.25±1.84	4.03	3.7	4.47
Serum urea	124.14±42.15	123	97.75	145.75
Serum sodium	134.70±4.80	136	131.5	138
Serum potassium	5.08±5.76	5.24	4.8	5.77
Serum chloride	97.17±5.21	97.3	93.93	99.75
Serum phosphorus	5.42±1.97	5.42	4.22	6.36
estimated glomerular filtration rate (eGFR)	6.00±3.00	5	4	7

Regarding the correlation between serum and salivary creatinine levels, a very weak positive linear relationship was observed with no significant association between them (p = 0.52) (Table 3).

**Table 3:** Correlation between serum creatinine and salivary creatinine levels "r" denotes the Pearson correlation coefficient, which measures the strength and direction of the linear link between two continuous variables. The scale goes from -1 to 1, with 1 indicating a perfect positive linear relationship, -1 a perfect negative one, and 0 no linear relationship. The "p-value" (p) is the likelihood of detecting the estimated correlation coefficient (or a more extreme value) if the population's true correlation coefficient is zero. A correlation coefficient with a low p-value (p < 0.05) is statistically significant.

Variables	Mean±SD	‘r’ value	p-value
Serum creatinine	9.50±3.05	0.093	0.52
Salivary creatinine	0.83±0.60		

Table 4 shows a statistically significant, moderately negative linear correlation exists between serum urea and serum albumin (r = -0.36; p = 0.01). Additionally, there is a statistically significant weak negative linear correlation between serum chloride and serum urea (r = -0.3; p = 0.03). Comparing serum chloride and serum sodium reveals a statistically significant, moderately positive linear relationship (r = 0.4; p = 0.004). Serum phosphorus and serum creatinine display a statistically significant moderate positive linear relationship (r = 0.44; p = 0.001). Moreover, eGFR and serum creatinine exhibit a statistically significant strong negative linear correlation (r = -0.79; p < 0.001), while eGFR and serum phosphorus demonstrate a statistically significant weak negative linear correlation (r = -0.30; p = 0.03).

**Table 4:** Correlation between serum creatinine and other specific lab results P stands for p value and if less than 0.05 is significant. N stands for the sample size which is 50. The Pearson correlation coefficient ranges from -1 to +1, where a value of +1 indicates a perfect positive linear relationship, meaning that as one variable increases, the other variable also increases proportionally. A value of -1 indicates a perfect negative linear relationship, meaning that as one variable increases, the other variable decreases proportionally. A value of 0 indicates there is no linear relationship between the variables.

Correlation Matrix for Serum Parameters		Serum Creatinine	Salivary Creatinine	Serum Calcium	Serum Albumin	Serum Urea	Serum Sodium	Serum Potassium	Serum. Chloride	Serum Phosphorus
Salivary Creatinine	Pearson Correlation	0.093								
	P	0.52								
	N	50								
Serum Calcium	Pearson Correlation	-0.124	-0.261							
	P	0.39	0.068							
	N	50	50							
Serum Albumin	Pearson Correlation	-0.096	-0.223	-0.05						
	P	0.507	0.12	0.731						
	N	50	50	50						
Serum Urea	Pearson Correlation	0.155	0.11	0.25	-0.359					
	P	0.281	0.448	0.079	0.01					
	N	50	50	50	50					
Serum Sodium	Pearson Correlation	0.186	0.121	0.226	0.097	0.025				
	P	0.196	0.404	0.114	0.505	0.865				
	N	50	50	50	50	50				
Serum Potassium	Pearson Correlation	-0.097	-0.158	-0.051	-0.019	-0.124	-0.051			
	P	0.502	0.272	0.727	0.897	0.393	0.724			
	N	50	50	50	50	50	50			
Serum Chloride	Pearson Correlation	0.11	0.202	-0.275	0.022	-0.3	0.404	0.004		
	P	0.446	0.16	0.053	0.88	0.034	0.004	0.979		
	N	50	50	50	50	50	50	50		
Serum Phosphorus	Pearson Correlation	0.444	0.241	-0.04	0.004	0.298	0.155	-0.036	0.088	
	P	0.001	0.092	0.783	0.979	0.035	0.283	0.802	0.542	
	N	50	50	50	50	50	50	50	50	
estimated glomerular filtration rate (eGFR)	Pearson Correlation	-0.79	-0.073	0.107	0.108	-0.186	-0.03	0.143	0.076	-0.301
	P	< 0.001	0.615	0.458	0.454	0.196	0.838	0.32	0.598	0.034
	N	50	50	50	50	50	50	50	50	50

## Discussion

Chronic kidney disease is a significant non-communicating disease contributing to poor health outcomes [10]. Elevated urea, creatinine, potassium, phosphorus, uric acid, and decreased calcium are the biochemical parameters estimated repeatedly in the management of these patients [11]. The kidneys excrete creatinine, a waste product of muscle breakdown, and CKD patients require frequent assessment of serum creatinine, resulting in multiple phlebotomies that damage the veins [6, 11].

This study examined 50 subjects admitted to the hospital with confirmed chronic kidney disease (CKD). The research aimed to explore an alternative, non-invasive method for evaluating creatinine levels in CKD patients. The serum creatinine values obtained ranged from 7.26 mg/dl to 12 mg/dl, with a median of 9.72 mg/dl. Salivary creatinine levels ranged between 0.45 mg/dl and 0.98 mg/dl, with a median of 0.75 mg/dl for this study.

Serum creatinine and salivary creatinine levels showed a weakly positive correlation (r = 0.093), but this correlation was not statistically significant (p = 0.52). Several factors may have contributed to this weak correlation, including the absence of CKD stage categorization, the timing of sample collection, the small sample size, and the time gap between the two tests, which involved a 90-minute fasting period before collecting the salivary sample. Additionally, the study did not account for testing samples (blood and saliva) before hemodialysis, which could have influenced the study's outcome. Changes in saliva flow rate and composition may also have affected creatinine concentration, potentially reducing its correlation with serum levels.

Nagarajan Bhuvaneswari et al.'s study similarly found no correlation between serum and salivary creatinine levels [11]. However, they observed a significant difference in salivary creatinine levels before and after dialysis, indicating a decrease following dialysis without a significant correlation to serum levels. Conversely, Xia et al.'s study reported a positive correlation between salivary and serum creatinine levels in both cases and controls, while Lloyd et al.'s study found a positive correlation only in CKD patients, not in controls [12, 13].

Upon further diagnostic analysis, it was revealed that serum urea and serum albumin exhibit a significant negative linear correlation (r = -0.36; p = 0.01). Additionally, there is a significant but weak negative linear correlation between serum chloride and serum urea (r = -0.3; p = 0.03). Moreover, a statistically significant moderately positive linear relationship was found between serum chloride and serum sodium (r = 0.4; p = 0.004), while serum phosphorus and serum creatinine displayed a statistically significant moderate positive linear relationship (r = 0.44; p = 0.001). Furthermore, a strong negative linear correlation was identified between eGFR and serum creatinine (r = -0.79; p = 0.001), and a weak negative linear significant correlation was observed between eGFR and serum phosphorus (r = -0.30; p = 0.03). These findings are consistent with those reported in previous studies [13, 14, 15].

Due to its large molecular size and limited lipid solubility, creatinine is unable to pass through membranes or tight junctions to enter saliva in healthy individuals [16]. However, in patients with CKD, there are theories suggesting that creatinine may diffuse into saliva due to altered cell permeability [16]. Nevertheless, our study lacked control groups and did not observe a significant presence of creatinine in saliva. Furthermore, the bacterial enzyme urease in the oral cavity breaks down urea, making it impractical to assess urea levels in saliva [16]. Therefore, oral hygiene practices heavily influence urease levels in saliva. Factors such as teeth brushing, gum chewing, eating, drinking, and oral hygiene maintenance can introduce biases in creatinine levels, as poor oral hygiene may lead to bleeding and falsely elevated saliva creatinine levels [16]. Additionally, nicotine and tobacco use can modify salivary composition and affect test results [17].

Limitations

The constraints identified in this study can serve as valuable insights for improving future research endeavors. This study was conducted at a single center with a relatively small sample size of 50 participants. A multicenter study with a larger sample size must be done to prevent the risk of skewed data. Also, this study lacked healthy control subjects, did not perform CKD staging, and did not compare pre- and post-hemodialysis samples. Furthermore, the time gap between the two tests did not account for the 90-minute fasting period required before collecting salivary samples.

## Conclusions

This study identified a slightly positive correlation between salivary and serum creatinine levels, although it did not reach statistical significance. However, this finding underscores the need for further investigation into the relationship between these two biomarkers. Multicentric studies with larger sample sizes are needed to clarify the significance and potential correlation between serum and salivary creatinine. Such endeavors are crucial for advancing our understanding of non-invasive biomarker assessment in clinical settings and could ultimately enhance diagnostic and monitoring approaches for conditions such as chronic kidney disease.
